# Direction-of-Arrival Estimation Methods in Interferometric Echo Sounding

**DOI:** 10.3390/s20123556

**Published:** 2020-06-23

**Authors:** Piotr Grall, Iwona Kochanska, Jacek Marszal

**Affiliations:** Faculty of Electronics, Telecommunications and Informatics, Gdansk University of Technology, Narutowicza 11/12, 80-233 Gdansk, Poland; iwokocha@pg.edu.pl (I.K.); jacek.marszal@pg.edu.pl (J.M.)

**Keywords:** direction-of-arrival, swath bathymetry, echo sounder, phase difference measurements, Prony’s method

## Abstract

Nowadays, there are two leading sea sounding technologies: the multibeam echo sounder and the multiphase echo sounder (also known as phase-difference side scan sonar or bathymetric side scan sonar). Both solutions have their advantages and disadvantages, and they can be perceived as complementary to each other. The article reviews the development of interferometric echo sounding array configurations and the various methods applied to determine the direction-of-arrival. “Interferometric echo sounder” is a broad term, applied to various devices that primarily utilize phase difference measurements to estimate the direction-of-arrival. The article focuses on modifications to the interferometric sonar array that have led to the state-of-the-art multiphase echo sounder. The main algorithms for classical and modern interferometric echo sounder direction-of-arrival estimation are also outlined. The accuracy of direction-of-arrival estimation methods is dependent on the configuration of the array and external and internal noise sources. The main sources of errors, which influence the accuracy of the phase difference measurements, are also briefly characterized. The article ends with a review of the current research into improvements in the accuracy of interferometric echo sounding and the application of the principle of interferometric in other devices.

## 1. Introduction

**Figure 1:**
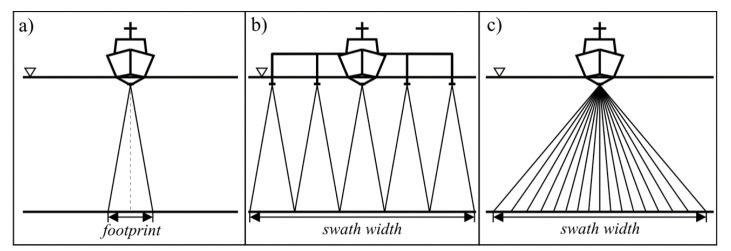
Cross track bottom coverage of various echo sounder systems. (**a**) Single-beam echo sounder. (**b**) Multi-channel (multi-transducer) echo sounder. (**c**) Multi-beam echo sounder. ∇ —water level. Swath (bottom strip personified by a single ping).

Acoustic techniques for surveying the sea bottom have been continuously developing since the early years of the 20th century. After WWII, the single beam echo sounder (SBES) started to become standard equipment on most merchant ships, as it facilitated safe navigation at sea [1]. The SBES uses a simple echolocation method that sounds the sea depth directly below the acoustic transducer of a small area, the so-called seabed footprint (Figure 1a). This records the depth profile along the path travelled by the vessel. Regardless of the success and widespread use of SBES, the demands for precise, high-resolution charting required more efficient means of surveying the sea [2,3]. To meet these requests, three categories of bathymetric survey systems have been designed:

1. The multi-channel echo sounder (MCES).
2. The multi-beam echo sounder (MBES).
3. The interferometric echo sounder (IES).

The MCES is a straightforward modification of the SBES (Figure 1b) [4]. It consists of several acoustic transducers mounted on horizontal booms at each side that operate in a synchronized manner. Nowadays, this solution is still used in rivers and in areas where the application of other systems is not economically justified or impractical. Instead, the MBES is the standard surveying tool (Figure 1c) [5]. It utilizes a wide fan of electronically-steered beams that allow for a wide strip of sea bottom to be simultaneously scanned. The MBES achieves the highest accuracy requirements as defined by the International Hydrographic Organization (IHO) [6]. Nevertheless, it has two main limitations: high purchase cost and a rapid decrease of accuracy for large angles of incidence. The third type of bathymetric system, the IES, was developed alongside the MBES in order to overcome these main limitations [2,3]. Initially, interferometric echo sounders were used in scientific research applications. In the mid 1990s, the IES reached a level of advancement that allowed it to be offered as a complete, off-the-shelf solution. At the beginning of the current millennium, the interferometric echo sounder started to be a viable alternative to the MBES [7]. Its angular coverage is usually greater than that of the MBES, which makes it more effective in shallow waters. The following sections present a brief description of the subsequent stages of the development of interferometric echo sounder array configurations. Factors affecting the accuracy of phase difference measurements are also briefly explained. The last section focuses on the current trends in research in the field of interferometric echo sounding.

## 2. Classical Interferometry

**Figure 2:**
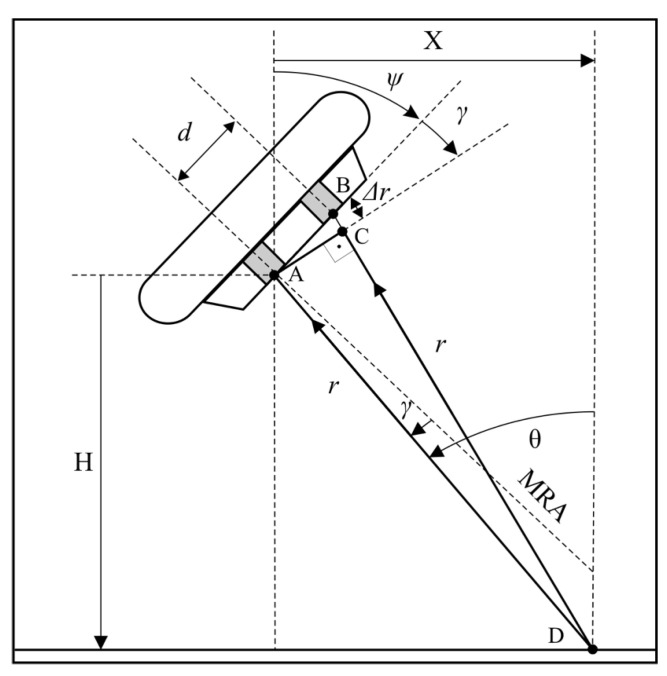
Interferometric echo sounder measurement principle.

Acoustic interferometry is a method of the measurement of physical properties inferred from the properties of the combined interaction of two or more acoustic waves. The most famous example of interference, though for electromagnetic waves, is Young’s double slit experiment [8]. If the distance between two slits is known and the distance between the maxima of the interference fringes is measured, the distance between the slit and the screen can be inferred. This inversion of the Young experiment lays the foundations for interferometric techniques and classical acoustic interferometry (Figure 2). First, assume that point D, situated on the bottom of the body of water, is the source of the echo signal. The distance form this point to receive elements A and B is different (r and r+Δr, respectively). If r>>d, the value Δr, from the right triangle ABC, can be calculated:

(1) dsin(γ)=Δr

where d is the distance between receive elements and γ is the direction-of-arrival (DOA) in relation to the receive array maximum response axis (MRA).

**Figure 3:**
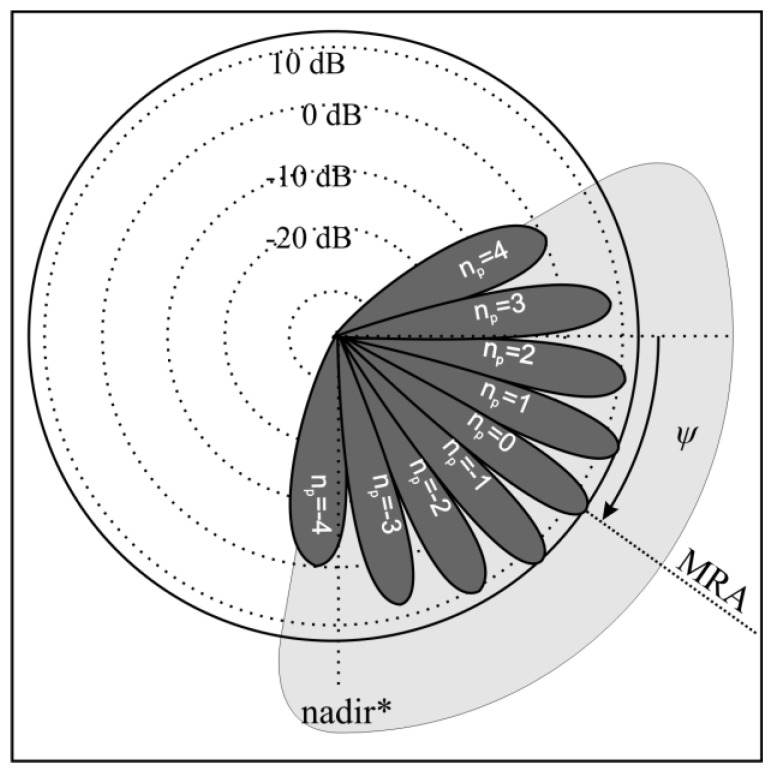
Interferometric echo sounder transmit and receive beam patterns. Light gray—transmit beam-pattern (simplified). Dark gray—receive beam-pattern. * nadir—the point directly below a particular place, the opposite of the zenith).

**Figure 4:**
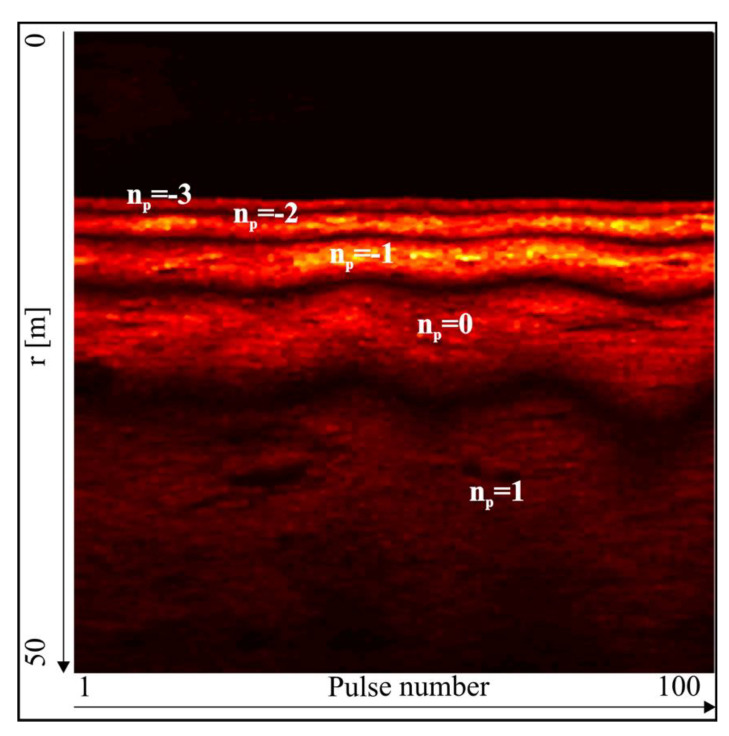
Interferometric fringes identification example. Image was generated using the raw signal from an EdgeTech 6205 system. d=4.5λ, H=13 m, and ψ=35°. Bottom configuration—flat. $r_{0}\approx H/\sin(\psi )\approx 30\;m.$ The fringes’ centers (the brightest parts of the fringes) indicate the maxima positions. The wave pattern on the interferogram was caused by the platform rolling.

In the interferometric echosounder, the value γ is sought based on the interference pattern obtained by combining the signals from two receive elements [3]. At first, the transmit element generates a wide beam impulse (Figure 3). After being reflected from the bottom, the impulse echo propagates back to a pair of identical receive elements. The distance between receive elements d is fixed and usually equals several wavelengths (see Table 1, positions 1 and 2). The electrical signal from the output of the receive elements is amplified and summed. The amplitudes are printed on thermal paper as a function of range (Figure 4). One vertical line of the printout represents one transmitted impulse. The height of the echogram is equivalent to the selected observation range. Adjacent lines form the interferogram. The interference fringes are equivalent to those in Young’s double slit experiment, and the interference pattern is used to assess the value of Δr in Equation (1).

The number of fringes and their locations in the interferogram depend on d, the characteristics of the transmit and receive elements, array tilt angle, depth, and selected observation range (Figure 4). Each maximum is related to the difference of acoustic path Δr, which is equal to an integer number of wavelengths λ, and Equation (1) transforms into [3]:

(2) $$d\sin\left(\gamma_{n_{p}}\right)=n_{p}\lambda$$

where $\gamma_{n_{p}}$ is the direction-of-arrival assigned to the given maximum, $n_{p}$ is the integer number assigned to the maximum, and λ is the acoustic wavelength at the receive elements.

Each maximum, for a given transmit impulse, is assigned to the distance r, which is equivalent to the two-way propagation time of the acoustic impulse in the water. This way, pairs of polar coordinates $\left(r_{n_{p}},\;\gamma_{n_{p}}\right)$ are obtained. The beginning of this coordinate system is located at the reference receive element (A in Figure 2). Its axis is aligned with the MRA. If array tilt angle ψ is known, the location of the echo source can be determined (see Figure 2):

(3) X=rsinθ=rsin(ψ+γ)

(4) H=rcosθ=rcos(ψ+γ)

where H is the depth below the receive array, X is the cross-track distance from the receive array, ψ is the array tilt angle in relation to the vertical, θ is the angle of incidence, and r is the distance from echo source to receive element A.

**Figure 5:**
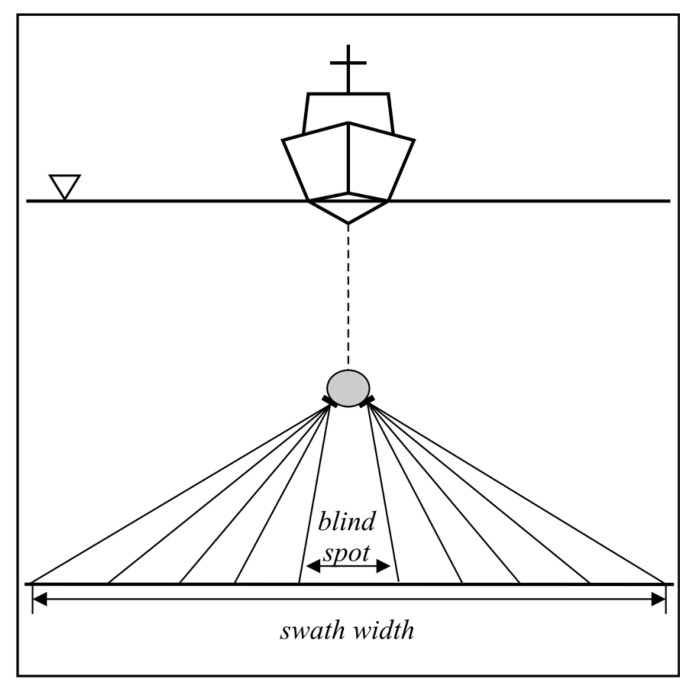
Towed classical/differential interferometric echo sounder cross track bottom coverage.

The advantage of the classical interferometric echo sounder is undoubtedly its simple design. Using only two receive elements, it can produce bottom estimates of good quality. This technique was therefore suitable for the general survey of large, deep areas such as the ocean’s abyss. Its heads (one at each side) were usually towed below the surface, which limited the impact of surface reflections (Figure 5). The main disadvantage of the interferometric echo sounder is its vulnerability to interfering signals coming from directions other than the sea bottom [14]. Classical interferometry, by principle, can only determine one DOA at a time. Other sources of echoes introduce biases in the determined direction. Another disadvantage is the fact that classical interferometry requires the manual and time-consuming post-processing of the interferograms to find the maxima and assign a number and range to each image strip [15]. Another minor limitation is that the angular separation between subsequent fringes varies because Equation (2) is nonlinear. Though towing the sonar heads away from the sea surface improves the accuracy of depth determination, this solution requires a layback calculation system to correctly georeference the results and assign final depths (corrected by the array draft). Another minor disadvantage is the need to find the $n_{p}=0$ fringe. To properly identify its location, approximate depth and tilt angle data are required (see the caption below Figure 4). An initial depth estimate can be obtained from a single-beam echo sounder, and the current tilt angle is the sum of the mounting tilt angle and the roll angle (registered by the on-board motion sensor).

## 3. Differential Interferometry

Classical interferometric methods are based on the analog signal processing scheme, but the development of fast digital processors and new transducer technologies have allowed for the application of digital processing to interferometric echo sounding. To facilitate this, the received signal is transformed inside the receiver into its analytic representation. Using I/Q decoding, the in-phase and quadrature components are calculated to enable the extraction of the signal instantaneous amplitude and phase [16]:

(5) $$A_{n}\left(t\right)=\left|s_{n}\left(t\right)\right|=\sqrt{x_{n}{\left(t\right)}^{2}+y_{n}{\left(t\right)}^{2}}$$

(6) $$\phi_{n}\left(t\right)=\arg\left(s_{n}\left(t\right)\right)=\arctan\left(\frac{y_{n}\left(t\right)}{x_{n}\left(t\right)}\right)$$

(7) $$s_{n}\left(t\right)=x_{n}\left(t\right)+j\;y_{n}\left(t\right)$$

where $s_{n}\left(t\right)$ is the analytic (complex) signal at *n*th receiver, $A_{n}\left(t\right)$ is the signal instantaneous amplitude, $\phi_{n}\left(t\right)$ is the signal phase, x(t) is the in-phase signal component, arg is the complex number argument, and y(t) is the quadrature signal component.

For any given moment *t*, the phase difference between two receive elements can be calculated [17]:

(8) $$\Delta \phi_{12}\left(t\right)=\phi_{1}\left(t\right)-\phi_{2}\left(t\right)$$

In practice, using properties of complex numbers, the phase difference is calculated using the following formula [18]:

(9) $$\Delta \phi_{12}\left(t\right)=\arg\left(s_{1}(t)\cdot s_{2}^{*}(t)\right)$$

where ${}^{*}$ denotes complex conjugate. The DOA was calculated from [16]:

(10) $$\gamma =\arcsin\left(\frac{\Delta \varphi_{12}+2\pi n_{p}}{kd}\right),\;n_{p}=\ldots ,-2,-1,\;0,\;1,\;2,\;\ldots ,$$

where k is the wavenumber (k=2π/λ). When d≤λ/2, the DOA is calculated unambiguously. If d>λ/2, phase ambiguity exists and assigning each measured difference to a certain $n_{p}$ value is required (analogously to classical interferometry).

**Figure 6:**
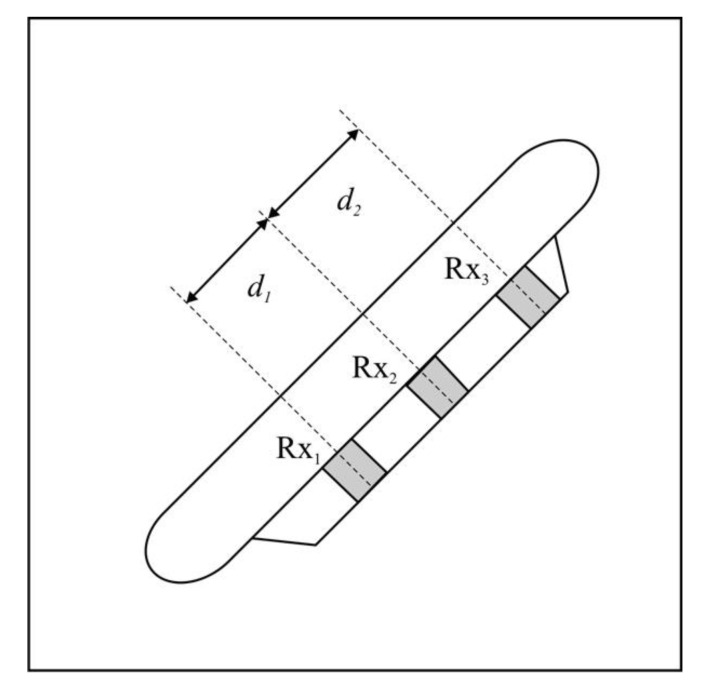
Vernier interferometer receive array configuration.

The Vernier method might be applied to help to resolve phase ambiguity [19]. It requires an additional receive element, however. The extraneous element is used to form two pairs that differ slightly in distance d (Figure 6). The phase difference is calculated from [11]:

(11) $$\Delta \phi_{V}\left(t\right)=\phi_{12}\left(t\right)-\phi_{23}\left(t\right),$$

where the values $\phi_{12}\left(t\right),\;\phi_{23}\left(t\right)$ can be calculated using Equations (8) or (9). The phase difference is limited to 〈−2π, 2π〉. In the case of low noise, the vast majority of the calculated differences lie within 〈−π, π〉 and the ambiguity is eliminated. When $\left|d_{1}-d_{2}\right|>\lambda /2$, ambiguity still remains, but even then, the number of fringes to resolve is greatly reduced or results for n≠0 can simply be rejected [20].

Difference interferometry was widely used for surveying the bottom of the oceans. Scientific research systems such as SeaMARC and TOPO-SSS used towed arrays [11] (see Table 1). Another method to limit the signals from undesired directions is the application of specially desired baffles [14]. The advantage of difference interferometry over classical interferometry is the improvement in the number of bottom samples. The number of samples depends on the signal sampling frequency and the maximum observation range. Similarly to classical interferometry, good quality data are obtained for large angles of incidence. The number of samples is usually less than 5000 per side per pulse, which provides high resolution survey data.

The disadvantage of this solution is the requirement of a more sophisticated digital receiver with high processing power to produce real-time results. Initially, the receive signals were recorded and analyzed in post-processing [21]. Though difference interferometry eliminated many of the problems of classical interferometry, it was still unable to resolve more than one DOA at a time. This limited the shallow-water applicability of the solution, as the platform has to be towed away from the surface of the water. Another disadvantage is its poor performance in the vertical direction [11]. In fact, the coverage of bathymetry is equivalent to that of the side scan sonar, which has a blind spot in the nadir area, as hardly any reliable data can be obtained from this direction.

## 4. Multi-Phase Difference Interferometry

**Figure 7:**
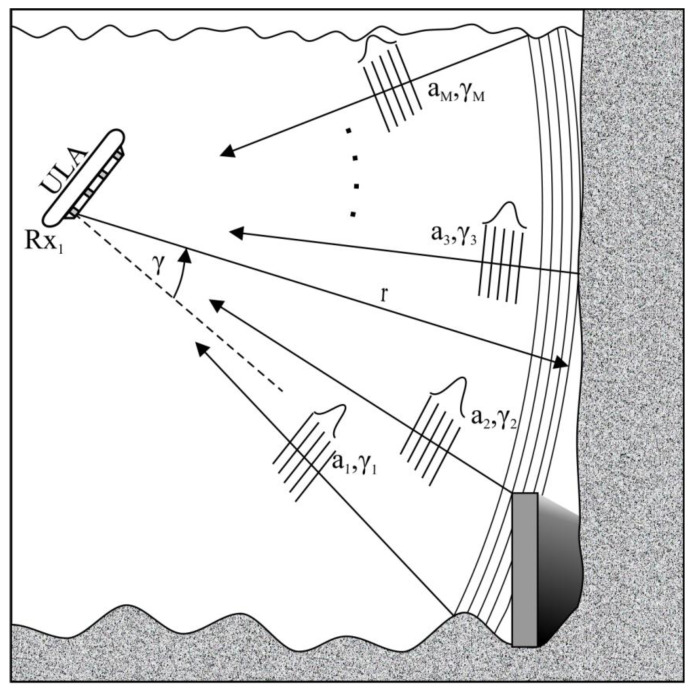
Generic multiphase echo sounder survey environment.

The multi-phase echo sounder (MPES) also known as the phase differencing side scan sonar (PDSS) or multi-phase difference interferometry (MPDI) is another step in the evolution of the interferometric echo sounder. It was designed to overcome main limitations of previous interferometric solutions, such as the capacity of one DOA at a time. It uses computed angle-of-arrival transient imaging (CAATI), which assumes the following (Figure 7) [22,23,24,25]:

1 A linear N -element equispaced array (uniform linear array—ULA) is used to measure transmitted signal echoes propagating in the same plane as the array.
2. At each instant in time, exactly M -independent, coplanar plane waves are incident on the receiving array.
3. The acoustic backscatter is narrowband.
4. The receiving array element output signals are in a steady state across the entire array.

What is more, elements are located at distances of less than half wavelength to avoid phase ambiguity. According to the above assumptions, at each receive element, the signal can be written as (generic formulation, time dependence of samples is omitted for clarity) [23]:

(12) $$\begin{array}{l} s(n)=\sum_{i=1}^{M}a_{i}\;e^{\left(\alpha_{i}+j\;u_{i}\right)\;d\left(n-1\right)}+w(n)\\ a_{i}\;=A_{i}e^{j\Theta_{i}^{}},\;u_{i}=k\;\sin\gamma_{i}\;,k=\frac{2\pi }{\lambda },\;n=1,2,\cdots ,N \end{array}$$

where $A_{i}$ is the signal amplitude at the reference receive element, $\Theta_{i}$ is the signal phase at the reference receive element, λ is the acoustic wavelength, $u_{i}$ is the spatial wavenumber, *α_i_* is the exponential damping factor, w(n) is noise.

In most practical applications, exponential damping factors α_i_ are assumed to be zero. From N signal samples, a set of linear backward–forward equations can be formed (forward-only or backward-only formulation is also possible) [22,23,26]:

(13) $$\left[\begin{array}{cccc} s\left(L\right) & s\left(L-1\right) & \cdots & s\left(1\right)\\ s\left(L+1\right) & s\left(L\right) & \cdots & s\left(2\right)\\ \vdots & \vdots & \ddots & \vdots \\ s\left(N-1\right) & s\left(N-2\right) & \cdots & s\left(N-L\right)\\ ---- & ---- & ---- & ----\\ s^{*}\left(2\right) & s^{*}\left(3\right) & \cdots & s^{*}\left(L+1\right)\\ s^{*}\left(3\right) & s^{*}\left(4\right) & \cdots & s^{*}\left(L+2\right)\\ \vdots & \vdots & \ddots & \vdots \\ s^{*}\left(N-L+1\right) & s^{*}\left(N-L+2\right) & \cdots & s^{*}\left(N\right) \end{array}\right]\left[\begin{array}{c} g_{1}\\ g_{2}\\ \vdots \\ g_{L} \end{array}\right]=-\left[\begin{array}{c} s\left(L+1\right)\\ s\left(L+2\right)\\ \vdots \\ s\left(N\right)\\ ----\\ s^{*}\left(1\right)\\ s^{*}\left(2\right)\\ \vdots \\ s^{*}\left(N-L\right) \end{array}\right]$$

or briefly in the vector/matrix notation:

(14) Ag=−h

From Equation (14), vector g is calculated, and its elements $g_{l}$ are used as coefficients of the polynomial equation:

(15) $$H_{t}(z)=1+\sum_{k=1}^{L}g_{k}z^{-k}=0$$

where $H_{t}(z)$ is the transfer function of the associated linear filter [26]. Complex zeros of Equation (15) are related to the sought directions by the relation [23]:

(16) $$z_{i}=e^{\left(\alpha_{i}+ju_{i}\right)d}$$

The direction can therefore be calculated from (compare Equation (10)):

(17) $$\gamma_{i}=\arcsin\left(\frac{\arg(z_{i})}{kd}\right)$$

Once directions $\gamma_{i}$ are determined, amplitudes $a_{i}$ can be calculated by solving another set of equations [23]. The aim of this technique is to extract unknown parameter pairs ($a_{i},\;\gamma_{i}$). However, this generally requires N>2M receive elements. In this solution, echoes not originating from the bottom are treated as additional unknown signals. The basic approach utilizes the least-squares solution to Equation (14) for L=M. Extraneous echoes, not originating from the bottom, are rejected in the filtering process [23].

The interferometric and multibeam echo sounder are both used for bottom mapping, but they are based on different measuring principles. The difference can be best described by the approach to Equations (3) and (4). The interferometric echo sounder determines angle (-s) γ for the given range r, while the MBES resolves range r within the predefined directions γ (beams).

While the MBES usually produces 200–400 beams (measuring points) per ping, the IES usually produces 8000–10,000 measuring points per ping (total of two heads). Nevertheless, a single-point measurement is generally noisier, meaning it has a greater depth variance than a single-point measurement of the MBES. Therefore, data reduction and generalization techniques have to be used to produce less measuring points for further processing (similar number of points to MBES processing). There is also a different distribution of measuring errors. While the accuracy of the IES is best around the crossing point of the MRA and bottom (see Section 4), the MBES is most accurate directly below the array, i.e., in the nadir zone. Some IES systems do not produce data in this blind spot, while others produce sparse, very noisy bottom samples in this area. The IES is a modification of side scan sonar, so it naturally produces a side scan sonar bottom image co-registered with bathymetry. Thanks to this, data processing and interpretation is much easier. The MBES lacks this feature. Generally the IES produces wider swaths than the MBES, especially for shallow water, which can significantly reduce survey time. For a depth range of 2–20 m, the width of the acceptable swath usually equals 8–12 times the depth below the transducer (H). MPDI may be directly attached to the hull of the survey vessel, which improves positioning accuracy, as the layback calculation system is no longer necessary. Currently, MPDI is capable of fulfilling the most stringent accuracy requirements and can provide real-time data [6,7,27]. Some of the systems struggle, however, with poor data in the nadir zone or no data at all in this direction.

## 5. Sources of Errors

The accuracy of the estimation of the DOA of an interferometric echo sounder, like any other measuring system, is dependent on various factors [17,18,28,29,30]. In the description provided below, we only focus on those specific to interferometric measurements. In the most basic propagation model, the accuracy of the measurement of the phase difference in Equation (8) is only dependent on the signal-to-noise ratio (SNR) (assuming a point-like coherent echo model and only two receive elements) [17]. This model, however, is too simplistic for the spatial and time variability of the echo signal received in an underwater environment and does not explain the observed phase difference variability [18].

**Figure 8:**
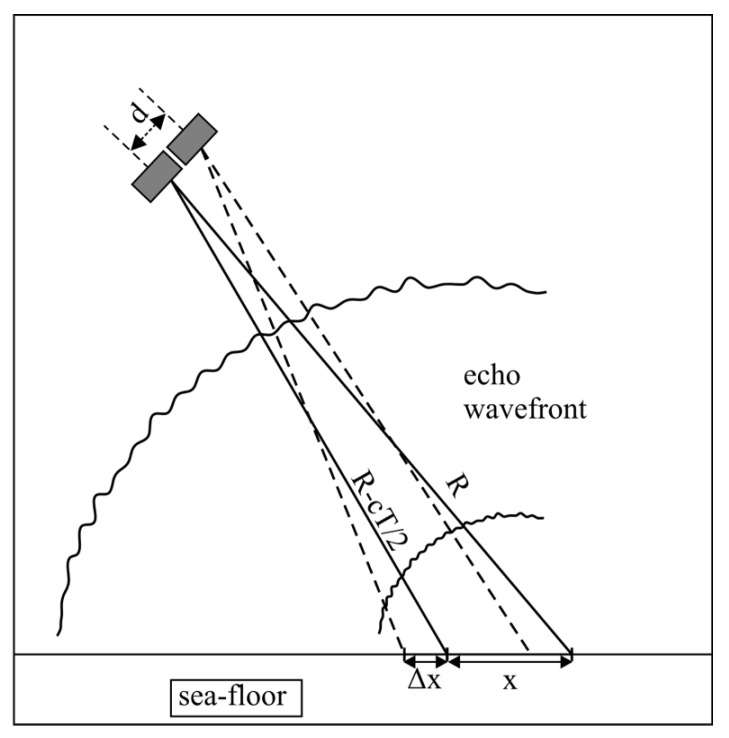
Shifting footprint and baseline decorrelation sources. x —footprint size; Δx—non-common footprints part; d—element spacing; T—pulse length; and c—speed of sound. Echo wavefront distortions are caused by non-coherent bottom scattering.

**Figure 9:**
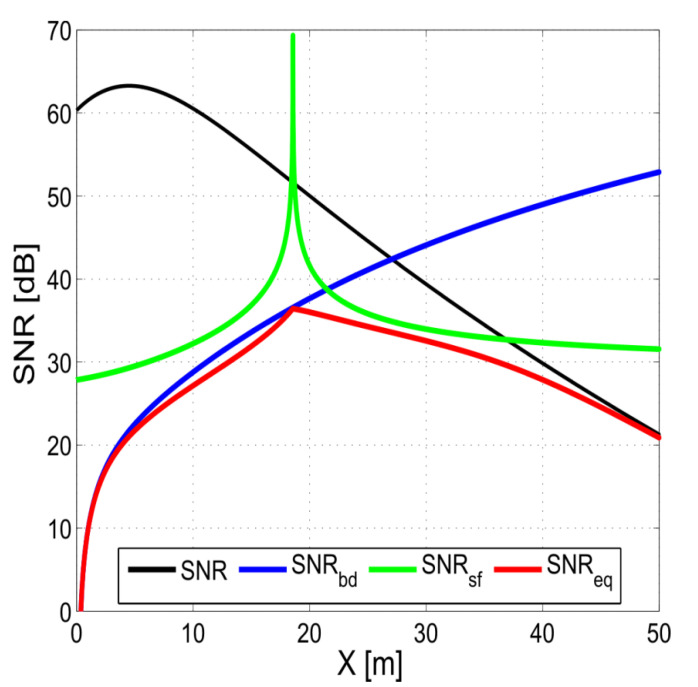
Influence of shifting footprint and baseline decorrelation on the equivalent signal-to-noise ratio (SNR). Example calculated for c = 1500 m/s, T = 1 ms, ψ=35°, H = 13 m, f = 500 kHz, and d = λ/2. SNR—nominal signal-to-noise ratio; $SNR_{bd}$—signal-to-noise ratio of the baseline decorrelation; $SNR_{sf}$—signal-to-noise ratio of the shifting footprint; and $SNR_{eq}$—equivalent signal-to-noise. $SNR_{sf}$, $SNR_{bd}$ and $SNR_{eq}$ are calculated based on the formulas found in [17], assuming a rectangle transmit pulse shape.

The spatial dimensions of the receive array and the finite size of the echo sources (acoustic footprints) result in inter-element coherence loss. This negative effect can be attributed to two main factors, i.e., the shifting footprint and baseline decorrelation (Figure 8) [18]. The shifting (sliding) footprint coherence loss is caused by the fact that for each receive element, the active footprint position is slightly different. The uncommon part of one footprint acts as noise for the other receiver signal, and only the common part of the footprints carries useful direction information. The shifting footprint effect is minimal around the MRA (Figure 9). Baseline decorrelation coherence loss is caused by the random distribution of the position, strength, and phase delay of the scatterers within the footprint. The resulting echo wavefront is not planar but is randomly distorted (Figure 8). These distortions gradually vanish with range, as the wavefront is smoothed. The impact of baseline decorrelation is greatest directly below the array (nadir zone) and diminishes with range (Figure 9). The element spacing in relation to the wavelength, array tilt, and bottom depth determine the actual impact of these two effects on accuracy [17].

The third type of noise is caused by volume reverberation that comes from random reflections from inhomogeneities in the water column. Reverberation can be treated as an additional range-dependent level of ambient noise, thus lowering the actual SNR. Additional sources of reflection, e.g., surface reverberation and multipath, lead to the degradation of the accuracy of those methods that assume only one source [18]. Generally, all noise sources cause signal decorrelation, which translates into the loss of the accuracy of the DOA estimation. Their impact on accuracy can be modelled by the notion of an equivalent SNR, which represents the global usable energy quota (i.e., carrying DOA information; see Figure 9) [17]. These sources of errors also apply to MPDI, which can be viewed as a superposition of several phase difference interferometers.

Another source of error for difference interferometry is caused by mistakes in the phase unwrapping operation [31]. The phase unwrapping errors are significantly limited by the application of the Vernier technique [32,33]. Nevertheless, they are inevitable when d>λ/2.

## 6. Current Research Review

Currently, many MPDI systems fulfill hydrographic survey accuracy requirements and are treated as equivalent to the MBES, especially in shallow waters applications [7,27]. However, there are still grounds for further research. First of all, the requirements might become more stringent, especially in the military and industrial areas of interest. Secondly, with the increase of the computational power of processing units, more computationally heavy methods might become available for real-time processing. Furthermore, other measuring devices might benefit from the application of interferometric echo sounding. Generally we can indicate three current areas of research into the interferometric echo sounding techniques:

1. Improvement in DOA accuracy.
2. The application of subarray processing.
3. The application of interferometric bathymetry to synthetic aperture sonar (SAS).

Improvements in the accuracy of interferometric echo sounding can be achieved in a number of ways. The most obvious one would be to increase the SNR by increasing the source level (of course, within the limit imposed by cavitation). However, once the SNR is above the decorrelation sources, a further increase in the source level will not provide any further improvement in accuracy (see Figure 9). Range and accuracy can also be improved by increasing the pulse length, but this also lowers the spatial resolution of the bottom image. A remedy to this range–resolution trade-off might be the application of FM pulses and matched filter processing [34,35,36]. The application of FM pulses lowers the impact of the shifting footprint. Unfortunately, increasing the effective pulse length unfavorably impacts baseline decorrelation. The net result is that FM can improve accuracy for low grazing angles, though not directly below the array. On the other hand, when using shorter pulses, the shifting footprint might become the limiting factor on accuracy.

The general trend outlined in Table 2 is the increase in the number of receive elements being utilized. In the case of difference interferometry, and one source of echo, multiple phase difference estimations during one time sample are being obtained, thus improving DOA estimation accuracy [37,38]. In the presence of multiple sources of echoes (a multipath, shallow water environment), MPDI accuracy is also being improved thanks to extraneous receive elements. Additionally, this increase also opens up the possibility of application of other than the least squares method outlined in Section 4, including subspace methods, with the possibility of achieving increased accuracy. From all the available methods to solve Equation (14), which are direct or indirect modifications of Prony’s method, the most representative examples are [26,39,40,41,42]:

1. Total least-squares.
2. Modified Prony.
3. Root-MUSIC.
4. ESPRIT-TLS.
5. Matrix Pencil.

Each method requires a different number of computations, and their immunity to modelling errors and noise levels is also different [41]. Usually, there is a compromise between the accuracy of the method and the number of computations required to achieve the final solution [42]. The above-mentioned high-resolution methods (including least-squares) also require the estimation of the number of signal echoes prior to the solution, i.e., model order (M) selection to perform subspace separation. However, standard procedures, such as the Akaike information criterion and the minimum description length methods [43], are not suitable for underwater acoustics. These methods assume a constant direction-of-arrival and do not take into account the inter-element coherence loss characteristic of the bathymetric sonar echo signals described in Section 4. As a result, these methods tend to overestimate the number of signal echoes. Recently, a new method for determining the number of signal echoes has been proposed by the authors, which is dedicated for this very purpose [27,44,45]. The proposed method takes inter-element coherence loss into account to apply a modified version of the matrix perturbation method [46]. This technique, although initially designed for the modified Prony method, might also be successfully applied to other model-based methods [27]. The application of subspace methods in conjunction with the proposed technique to determine the number of echo signals improves the accuracy of DOA estimation compared to the standard least-squares (LS) method. ESPRIT-TLS and root-MUSIC perform similarly but generally require a larger number of computations [27].

A further increase in the number of receiving elements allows for the application of a hybrid approach, called subarray processing [47,48]. First, initial bottom estimates are obtained, and then the array is divided into several overlapping subarrays that are steered (beamformed) in the direction of the initial bottom estimates. Finally, interferometric processing is applied to the outputs of the subarrays giving the final DOA estimation for each bottom sample. Beamforming partially suppresses signals from unwanted directions, thus limiting their negative impact on accuracy. Either a uniform and non-uniform linear array can be used in this solution [49,50]. A variant of this method is commonly used in the MBES to increase accuracy for low grazing angles. This variant uses two subarrays and the initial bottom estimates are obtained via beamforming [51].

With the advent of SAS, it was soon realized that interferometric techniques might be used to obtain depth estimates based on the high-quality sonar images it produces [52]. To obtain depth estimates, one or two rows of receive elements are placed above the original receive array [53,54]. Since SAS devices are mounted on underwater vehicles, the multipath echoes caused by proximity to the sea surface are absent, and simple—assuming one echo—difference interferometry or the Vernier technique might be used [55].

## 7. Conclusions

Changes in the DOA estimation methods applied in interferometric echo sounding reflect the increasing need to obtain more accurate and effective depth measuring devices. Modifications in array configuration have allowed for the application of more complex algorithms for DOA estimation, which, combined with advanced signal processing techniques, have helped to eliminate its main shortcomings. The multiphase echo sounder is expected to gain more interest in the following years due to its multiple advantages. Its higher efficiency at surveying in shallow waters and lower cost of manufacturing, as compared to the MBES, make it a reasonable choice for coastal waters and harbors. It is also a solution that is easily applied in underwater and surface unmanned autonomous vehicles due to the reduced array size. Constant demands for large amounts of high-quality bathymetric data and advances in the software and hardware for signal processing are expected to be the driving factor behind the development and proliferation of multiphase survey techniques in the years to come. It is also expected that subarray processing will gain more interest in the future because it is a solution that combines the advantages of both interferometric and multibeam processing.

## Figures and Tables

**Table 1:** Parameters of selected scientific research interferometric systems (classical and differential) [3,9,10,11,12,13].

No.	System Name (Prod. Year)	Freq. [kHz] ^1^	No. of Receive Elements	d	Beam Width [deg. × deg.] ^3^
1	Telesounding (1974)	250	1 ^2^ 2	33/60 λ 33 λ	1 × 50
2	Bathyscan (1982)	300	2	10 λ, 11 λ	1 × 25
3	TOPO-SSS (1982)	160	2	1.9 λ	2 × 45
4	SeaMARC II (1983)	11, 12	2	0.5 λ	2 × 55
5	SeaMARC/S (1985)	150	3	λ	2 × 45
6	SeaMARC/R (1989)	11, 12	2	0.5 λ	2 × 55
7	SYSTEM120 (1989)	120	3	λ	2 × 50
8	SeaMARC TAMU (1990)	11, 12	3	0.45 λ	2 × 65
9	SYSTEM09 (1990)	9, 10	2	0.8 λ	2.5 × 65
10	GLORI-B (1992)	6.8, 6.3	2	0.7 λ	2.7 × 35
11	Deepscan (1999)	60, 120	3	0.8 λ	1.5 × 50

^1^ Double values separated by a comma mean a different frequency for each side—port and starboard. ^2^ Mirror bottom reflection generated by a specially designed baffle. ^3^ Horizontal × vertical.

**Table 2:** Multi-phase difference interferometry (MPDI) systems. ^1^ (Source: system manuals, data sheets, and private correspondence).

No.	Manufacturer, System Name	Freq. [kHz]^1^	No. of Receive Elements	Beam Width [deg. × deg.]	
1	Klein, HydroChart 3500/5000	455	4/5	0.4 × 120	
2	ITER Systems, Bathyswath-2	117 234 468	4	0.85 0.55 0.55	× 140
3	Kongsberg, GeoswathPlus	125 250 500	4	0.85 0.55 0.55	× 140
4	Teledyne, Benthos C3D ^2^	200	6	1 × 100	
5	Edgetech, 4600	230 550	8	0.64 0.5	× 100
6	Edgetech, 6205/6205s	230 550	10	0.7 0.5	× 100

^1^ Element separation approx. 0.5 λ. ^2^ No longer in production.
